# Supplementary material for: Reaction-Diffusion Systems: Self-Balancing Diffusion and the Use of the Extent of Reaction as a Descriptor of Reaction Kinetics
Source: Int J Mol Sci. 2022 Sep 10;23(18):10511. doi: 10.3390/ijms231810511 (PMC9505240; doi:10.3390/ijms231810511)
Supplement: Supplementary file 1 [file ijms-23-10511-s001.zip › ijms-1790872-supplementary.pdf]

Supplementary material

Reaction-diffusion systems: self-balancing diffusion and the use of the extent of reaction as a descriptor of reaction kinetics

Miloslav Pekař

Brno University of Technology, Faculty of Chemistry, Purkyňova 118, 612 00 Brno, Czech Republic

E-mail: pekar@fch.vut.cz

Ad sec. 3. Theoretical background

For readers convenience, a brief but sufficiently comprehensive theoretical overview is given here (for details see [15, 16, 18]). The general mass balance of a chemically reacting mixture entails that in any fixed volume V of the mixture the sum of the masses of all constituents changes only by transport through the fixed volume surface ∂V:

$$\frac{d}{dt}\sum_{\alpha=1}^{n}\int_V \rho_\alpha \, dv + \sum_{\alpha=1}^{n}\int_{\partial V} \rho_\alpha \mathbf{v}_\alpha . \mathbf{n} \, da = 0. \tag{S1}$$

A list of symbols is attached at the end of the main text; here, we only stress that the composition is expressed using the mass density ρ_α, which is the mass of constituent α in unit volume of the whole mixture; in chemistry it is usually called the mass or weight concentration. All relevant quantities are considered as fields, that is, space and time dependent. Using Gauss's theorem, balance (S1) can be localized:

$$\sum_{\alpha=1}^{n}\frac{\partial \rho_\alpha}{\partial t} + \sum_{\alpha=1}^{n} \mathrm{div}\rho_\alpha \mathbf{v}_\alpha = 0. \tag{S2}$$

Finally, the local balance for constituent α is obtained:

$$\frac{\partial \rho_\alpha}{\partial t} + \mathrm{div}\rho_\alpha \mathbf{v}_\alpha = r_\alpha \tag{S3}$$

where the component masses produced (or consumed) by chemical reactions r_α (the reaction rates) fulfill the condition

$$\sum_{\alpha=1}^{n} r_\alpha = 0. \tag{S4}$$

The transformation of mass-based to molar-based quantities is given in section 3.3 of the main text. Using the definition of the density of the mixture $\rho = \sum_{\alpha=1}^{n} \rho_\alpha$, balance (S2) can be rewritten as

$$\frac{\partial \rho}{\partial t} + \sum_{\alpha=1}^{n} \mathrm{div}\rho_\alpha \mathbf{v}_\alpha = 0. \tag{S5}$$

Let us define the barycentric velocity $\mathbf{v}^w$ as the average velocity of the mixture based on the mass fraction w_α:

$$\mathbf{v}^w = \sum_{\alpha=1}^{n}(\rho_\alpha/\rho)\mathbf{v}_\alpha \equiv \sum_{\alpha=1}^{n} w_\alpha \mathbf{v}_\alpha. \tag{S6}$$

It follows from this definition that

$$\mathrm{div}\rho\mathbf{v}^w = \sum_{\alpha=1}^{n} \mathrm{div}\rho_\alpha \mathbf{v}_\alpha. \tag{S7}$$

Substituting from (S7) into (S5) and using the following definition of the material derivative relative to the barycentric velocity (symbolized by a dot, exemplified on density)

$$\dot{\rho} = \frac{\partial \rho}{\partial t} + \mathbf{v}^w . \operatorname{grad} \rho, \tag{S8}$$

we obtain the local mass balance for the whole mixture in the form:

$$\dot{\rho} + \rho \operatorname{div} \mathbf{v}^w = 0. \tag{S9}$$

The diffusion velocity $\mathbf{u}_\alpha^w$ is defined relative to the barycentric velocity:

$$\mathbf{u}_\alpha^w = \mathbf{v}_\alpha - \mathbf{v}^w. \tag{S10}$$

Then

$$\operatorname{div} \rho_\alpha \mathbf{v}_\alpha = \operatorname{div} \rho_\alpha \mathbf{u}_\alpha^w + \rho_\alpha \operatorname{div} \mathbf{v}^w + \mathbf{v}^w . \operatorname{grad} \rho_\alpha. \tag{S11}$$

The product $\rho_\alpha \mathbf{u}_\alpha^w = \mathbf{j}_\alpha^w$ is called the (mass) diffusion flux of component α; its units are kg m^{-2} s^{-1}. With the aid of (S8), (S9), (S11), and the definition of the mass fraction w_α already used in (S6)

$$w_\alpha = \rho_\alpha / \rho \tag{S12}$$

the component balance (S3) can be rewritten in the following form:

$$\rho \dot{w}_\alpha = -\operatorname{div} \rho_\alpha \mathbf{u}_\alpha^w + r_\alpha. \tag{S13}$$

The last equation is transformed in terms of the molar component reaction rate (J^α), which is more common in chemistry and chemical (reaction) engineering, using the constituents' molar masses M_α. We thus obtain:

$$\frac{\dot{w}_\alpha}{M_\alpha} = -\frac{1}{\rho M_\alpha} \operatorname{div} \rho_\alpha \mathbf{u}_\alpha^w + \frac{J^\alpha}{\rho}, \; \alpha = 1, \ldots, n \tag{S14}$$

where $J^\alpha = r_\alpha / M_\alpha$. The molar masses are components of the formal vector of molar masses $\mathbf{M}$ (units kg mole^{-1} in this work) located in an abstract n-dimensional vector space, called the mixture space, with a (covariant, n-dimensional) basis $\mathbf{e}^\alpha$ [15] (see also [18], p. 151-152):

$$\mathbf{M} = \sum_{\alpha=1}^n M_\alpha \mathbf{e}^\alpha. \tag{S15}$$

Let us define another vector (using a contravariant, n-dimensional, basis):

$$\boldsymbol{\omega} = \sum_{\alpha=1}^n \frac{w_\alpha}{M_\alpha} \mathbf{e}_\alpha \tag{S16}$$

(units of $\boldsymbol{\omega}$ are mole kg^{-1}). From the properties of the base vectors, it follows that

$$\mathbf{M} \,.\, \boldsymbol{\omega} = \sum_{\alpha=1}^n w_\alpha = 1. \tag{S17}$$

The balances (S14) can be written in the compact form

$$\dot{\boldsymbol{\omega}} = \boldsymbol{\sigma} + \boldsymbol{\omega}^+ \tag{S18}$$

where

$$\boldsymbol{\sigma} = -\sum_{\alpha=1}^n \frac{1}{\rho M_\alpha} (\operatorname{div} \rho_\alpha \mathbf{u}_\alpha^w) \, \mathbf{e}_\alpha \tag{S19}$$

and

$$\boldsymbol{\omega}^+ = \sum_{\alpha=1}^n \frac{J^\alpha}{\rho} \mathbf{e}_{\boldsymbol{\alpha}}. \tag{S20}$$

Both $\boldsymbol{\sigma}$ and $\boldsymbol{\omega}^+$ have units mole kg^{-1} s^{-1} and are expressed by (S19) and (S20) in the n-dimensional contravariant basis.

Due to the linear algebra of the permanence of atoms, the mixture space is divided into two orthogonal subspaces – one (of the dimension $h < n$) which has no special name and is denoted *W*, the other (of the dimension $n - h$) which is called the reaction space [15]. This division is an outcome of linear algebra of stoichiometry. The reaction rates and, thus, also the vector $\boldsymbol{\omega}^+$ lie in the reaction space. In contrast, the vector of molar masses is located in the subspace *W* [15] and from the orthogonality of the two subspaces it follows that

$$\mathbf{M} \,.\, \boldsymbol{\omega}^+ = 0. \tag{S21}$$

From (S17) it follows that $\mathbf{M} \,.\, \dot{\boldsymbol{\omega}} = 0$, and combining with (S18) and (S21) we obtain

$$\mathbf{M} \,.\, \boldsymbol{\sigma} = 0. \tag{S22}$$

This is a general condition of diffusion in chemically reacting mixtures, which restricts the (divergence of) diffusion fluxes (hidden in $\boldsymbol{\sigma}$).

Diffusion is called self-balancing [16] if the vector $\boldsymbol{\sigma}$ also lies in the reaction space. Then, the vector $\dot{\boldsymbol{\omega}}$ is also located in the reaction space [16]. Any vector from the reaction space is perpendicular to the basis of the subspace *W*, which we denote $\mathbf{f}_\sigma$ $(\sigma = 1, \dots, h)$. Consequently, we can formulate the following condition for diffusion to be self-balancing:

$$\boldsymbol{\sigma} \,.\, \mathbf{f}_\sigma = 0. \tag{S23}$$

Ad sec. 2.5. Note on generalized extents; summarizing notes

Rodrigues et al. [17] proposed a generalization of the concept of the reaction extent (and other extents in general) to distributed reaction systems (i.e., space-distributed systems with diffusion). Detailed comparison of their approach and the findings described in this work is beyond the scope of this paper and only several important points will be highlighted. Perhaps the main point is the difference in the definition of the reaction extent. The method described here defines the extent with respect to a fixed point (in time and space) and explicitly to a compositional variable – an approach common in chemistry and chemical engineering. Rodrigues et al. [17] define the extent of reaction implicitly with respect to certain formal concentration variables which change over time (and in space, generally), these changes are described by balance equations valid for non-reacting non-diffusing species.

As an example, let us consider a (single-phase) one-dimensional tubular reactor with its balance [17] written in simplified form as (the original symbols [17] were retained):

$$\frac{\partial \mathbf{c}}{\partial t} + \frac{\partial v_z \mathbf{c}}{\partial z} = \mathbf{N}^{\mathrm{T}} \mathbf{r} + \mathbf{r}_{\mathrm{d}} \tag{S24}$$

where $\mathbf{c}$ is the vector of molar concentrations; v_z is the advective velocity in the z-direction; $\mathbf{r}$ and $\mathbf{r}_{\mathrm{d}}$ are vectors of the reaction and diffusion rates, respectively; and $\mathbf{N}^{\mathrm{T}}$ is the transpose of the stoichiometric matrix. In fact, the component reaction rates are directly expressed in terms of reaction rates (and the corresponding stoichiometric coefficients) and this is another difference – in our work the reaction rates are not introduced in mass balance directly (a priori) but as a consequence of the linear algebra of the permanence of atoms, cf. the way to eq. (30) in the main text. The generalized extents of reactions, denoted by $\mathbf{x}_{\mathrm{r}}$ (vector of extents, in fact), are defined by the following equation [17]

$$\frac{\partial \mathbf{x}_{\mathrm{r}}}{\partial t} + \frac{\partial v_z \mathbf{x}_{\mathrm{r}}}{\partial z} = \mathbf{r} \tag{S25}$$

which, in fact, is a version of eq. (S24) for non-diffusing species. To reconstruct the initial system (S24), additional definitions and equations are necessary. Another extent – the generalized extent of diffusion $\mathbf{x}_\mathrm{d}$ – is introduced [17]. Thus,

$$\frac{\partial \mathbf{x}_\mathrm{d}}{\partial t} + \frac{\partial v_z \mathbf{x}_\mathrm{d}}{\partial z} = \mathbf{r}_\mathrm{d}. \tag{S26}$$

Similarly to (S25), this is a version of eq. (S24) for diffusing-only (non-reacting) species. Finally, a formal concentration variable $\mathbf{c}_\mathrm{b}$ fulfilling the equation

$$\frac{\partial \mathbf{c}_\mathrm{b}}{\partial t} + \frac{\partial v_z \mathbf{c}_\mathrm{b}}{\partial z} = \mathbf{0} \tag{S27}$$

is introduced [17]. Eq. (S27) is a reaction-less, diffusion-less version of (S24). The last item required for the reconstruction of the initial system (S24) is the relationship between all concentration and extent variables:

$$\mathbf{c} = \mathbf{N}^\mathrm{T}\mathbf{x}_\mathrm{r} + \mathbf{x}_\mathrm{d} + \mathbf{c}_\mathrm{b}. \tag{S28}$$

The initial system (S24) is obtained by combining (S27), (S25), (S26), and (S28). Extents can be calculated from eq. (S28) using the solution of eqs. (S24) and (S27) if there is an inverse of the transposed stoichiometric matrix. If there is no inverse the extents can still be calculated by solving (S25) or (S26). Thus, instead of a time-fixed reference point ($\boldsymbol{\omega}_0$), Rodrigues et al. [17] use a time-changing reference point $\mathbf{c}_\mathrm{b}$ to define the extent of reaction (and also of diffusion).

The approach of Rodrigues et al. [17] can be combined with the methodology presented in this paper by a (formal) splitting of the vector $\boldsymbol{\omega}$ into reaction and diffusion contributions:

$$\boldsymbol{\omega} = \boldsymbol{\omega}_\mathrm{r} + \boldsymbol{\omega}_\mathrm{d}. \tag{S29}$$

Defining

$$\dot{\boldsymbol{\omega}}_\mathrm{r} = \boldsymbol{\omega}^+,\ \dot{\boldsymbol{\omega}}_\mathrm{d} = \boldsymbol{\sigma}, \tag{S30}$$

using (7) from the main text we obtain

$$\dot{\boldsymbol{\omega}}_\mathrm{r} + \dot{\boldsymbol{\omega}}_\mathrm{d} = \boldsymbol{\omega}^+ + \boldsymbol{\sigma} = \dot{\boldsymbol{\omega}}. \tag{S31}$$

The equations under (S30) are analogs of (S25) and (S26). They seem to bring nothing new to the methodology of this paper. The vector $\boldsymbol{\omega}^+$ is always in the reaction space and therefore so is the vector $\dot{\boldsymbol{\omega}}_\mathrm{r}$. If the vector $\boldsymbol{\sigma}$ is not in the reaction space, then neither is $\dot{\boldsymbol{\omega}}_\mathrm{d}$ and the situation with introducing the extent of reaction with reference to a fixed point in time ($\boldsymbol{\omega}_0$) is the same as that without the splitting described in (S29).

On the other hand, equations (S30) can be utilized similarly as in [17] to define generalized extents, i.e. in (S25) and (S26), which is not within the scope of this work. The advantage of introducing generalized reaction ($\mathbf{x}_\mathrm{r}$) and diffusion ($\mathbf{x}_\mathrm{d}$) extents should consist in a clear indication of which concentration changes (of a component) are caused by reactions and which by diffusion. Yet, reaction-caused changes are already given by the rates of independent reactions, cf. eq. (S20) and text around eq. (30):

$$\dot{\boldsymbol{\omega}}_\mathrm{r} \equiv \boldsymbol{\omega}^+ = \sum_{\alpha=1}^{n} \frac{J^\alpha}{\rho} \mathbf{e}_{\boldsymbol{\alpha}} = \sum_{\alpha=1}^{n} \left(\frac{1}{\rho} \sum_{p=1}^{n-h} P^{p\alpha} J_p \right) \mathbf{e}_{\boldsymbol{\alpha}} \equiv \mathbf{J}. \tag{S32}$$

If the reaction rates are expressed explicitly as functions of concentrations, e.g. by the mass-action law common in kinetics, a model for the diffusion flux is employed (e.g. Fick's law), the differential equations of mass balance can be solved, concentrations at any time (and space point) can be obtained, and from them their time derivatives and the rates of independent reactions at any time can be calculated, giving the (rate of) change caused by reactions; the remaining change is the

result of diffusion, as eqs. (S14) or (42) also indicate: $\boldsymbol{\sigma} = \dot{\boldsymbol{\omega}} - \boldsymbol{\omega}^{+}$**.** The only difference is that the reaction rates (or diffusion fluxes) show actual rates of concentration changes, whereas the extents show changes relative to some reference. Thus, units of extent do not contain time units, as already noted below (6) in the main text. Concretely, the units of ξ based on (S16) are mol g^{-1}, the units of $\mathbf{x}_{\mathrm{r}}$ are mol m^{-3}, and the units of J_p are mol m^{-3} s^{-1}. Extents are thus useful when one is not satisfied with actual or instantaneous characteristics (rates) but, for a particular reason, prefers overall or integral quantities (extents). On the other hand, reaction rates can also be integrated using (34), giving an overall descriptor. Integration in (28) provides a combined (reaction plus diffusion) overall descriptor.

Note that eq. (S32) enables the compact balance form (42) to be expressed in terms of reaction rates:

$$\boldsymbol{\sigma} = \dot{\boldsymbol{\omega}} - \boldsymbol{\omega}^{+} = \dot{\boldsymbol{\omega}} - \mathbf{J} = \dot{\xi} - \mathbf{J} \qquad \text{(S33)}$$

(eq. (5) was used in the last equality). Eq. (S33) shows explicitly that (only) when there is no diffusion, (independent) reaction rates are directly equal to the time derivatives of the extents of these reactions.

References

Numbering from the main text retained here.

[15] Bowen, R.M. On the Stoichiometry of Chemically Reacting Systems. *Arch. Rational Mech. Anal*. **1968**, *29*, 114-124.

[16] Truesdell, C. *Rational Thermodynamics*, 2nd ed,; Springer:, New York, 1984; pp. 356-357.

[17] Rodrigues, D.; Billeter, J.; Bonvin, D. Generalization of the concept of extents to distributed reaction systems. *Chem. Eng. Sci*. **2017**, *171*, 558-575.

[18] Pekař, M.; Samohýl, I. *The Thermodynamics of Linear Fluids and Fluid Mixtures*, Springer: Cham, 2014.
